# Characterization of genetic diversity and gene mapping in two Swedish local chicken breeds

**DOI:** 10.3389/fgene.2015.00044

**Published:** 2015-02-17

**Authors:** Anna M. Johansson, Ronald M. Nelson

**Affiliations:** ^1^Department of Animal Breeding and Genetics, Swedish University of Agricultural SciencesUppsala, Sweden; ^2^Department of Clinical Sciences, Swedish University of Agricultural SciencesUppsala, Sweden

**Keywords:** chicken, SNP, comb color, genetic diversity, dermal hyperpimentation, EDN3

## Abstract

The aim of this paper is to study genetic diversity in the two Swedish local chicken breeds Bohuslän-Dals svarthöna and Hedemorahöna. The now living birds of both of these breeds (about 500 for Bohuslän-Dals svarthöna and 2600 for Hedemorahöna) originate from small relicts of earlier larger populations. An additional aim was to make an attempt to map loci associated with a trait that are segregating in both these breeds. The 60k SNP chip was used to genotype 12 Bohuslän-Dals svarthöna and 22 Hedemorahöna. The mean inbreeding coefficient was considerably larger in the samples from Hedemorahöna than in the samples from Bohuslän-Dals svarthöna. Also the proportion of homozygous SNPs in individuals was larger in Hedemorahöna. In contrast, on the breed level, the number of segregating SNPs were much larger in Hedemorahöna than in Bohuslän-Dals svarthöna. A multidimensional scaling plot shows that the two breeds form clusters well-separated from each other. Both these breeds segregate for the dermal hyperpigmentation phenotype. In Bohuslän-Dals svarthöna most animals have dark skin, but some individuals with lighter skin exists (most easily detected by their red comb). An earlier study of the Fm locus showed that this breed has the same complex rearrangement involving the *EDN3* gene as Silkie chicken and two other studied Asian breeds. In the breed Hedemorahöna, most individuals have normal skin pigmentation (and red comb), but there are some birds with darker skin and dark comb. In this study the involvement of the *EDN3* gene is confirmed also in Hedemorahöna. In addition we identify a region on chromosome 21 that is significantly associated with the trait.

## Introduction

There are 11 Swedish local chicken breeds. The conservation of the Swedish local chicken breeds is coordinated by the Swedish association for local poultry (Svenska Lanthönsklubben). The Swedish local chicken breeds originate from different parts of Sweden and only a small number of birds remained when they were rescued from extinction. An interesting aspect is that, although they have gone through severe bottle-necks, several of the breeds still retain much phenotypic diversity. This makes these breeds promising to use for mapping of the segregating traits. The genetic diversity is probably limited which will make the loci involved in the segregating traits easier to identify. Sequencing of the mtDNA D-loop in nine of these breeds showed limited diversity but revealed multiple maternal origins in several of the breeds (Englund et al., 2015). Due to the limited mtDNA diversity, it is necessary to analyze autosomal markers in these breeds, in order to study the relatedness between breeds. Analysis of a set of dense autosomal markers would also have the added value of giving the possibility to do genetic mapping of interesting traits in these breeds.

In this paper we chose to study the breeds Bohuslän-Dals svarthöna and Hedemorahöna. We chose these breeds since both breeds segregate for the dermal hyperpigmentation phenotype. We have chosen the comb color as phenotype to study, since this is an easy phenotype to accurately collect during the short visit to the chicken owners. Bohuslän-Dals svarthöna originates from the northern part of Bohuslän in western Sweden (close to the Norwegian border). Around 1899, a woman got the ancestors of the current population of this breed as a wedding gift. Her two sons inherited the flock and in 1958 a man got the birds from the two brothers (Olsson, 2004). These black birds is said to have be common in this area in old times and there is a legend stating that they are the descendants of birds that sailors brought from a foreign country. The current population size is approximately 500. Most individuals have black feathers but also black beaks, legs, skin, and dark colored meat. Some individuals with lighter skin exists (most easily detected by their red comb). The black pigmentation was recently shown to be due to the same complex rearrangement involving the *Endothelin 3* (*EDN3*) locus as Silkie chicken and two other studied Asian breeds (Dorshorst et al., 2011). The Hedemorahöna originates from the area close to Hedemora in the Dalarna county. They were rediscovered in the 1980s when a woman saw a flock that reminded her of the chickens her parent had during her childhood (Olsson, 2004). The population size is approximately 2600. Hedemorahöna have a wide range of plumage colors. Most individuals have normal skin pigmentation (and red comb), but some birds have been observed to have darker skin and dark comb.

What we have seen from our collected data, is that the comb color can be categorized into three categories: red, semi-dark, and dark. This has also been noted in several generations by the owners of these birds. It is not a continuous scale and it is easy to type each bird into one of these categories. Males have all three comb colors. However, females only have red or dark combs. This points to the involvement of the Z chromosome in the trait. Additional observations from a bird owner, who is interested in genetics, show that the inheritance of comb color can mostly be explained as a Z-linked inheritance, but that there are exceptions that do not fit with a single Z-linked locus (Thomas Englund, Pers. Commun.). The *Fm* and *id* loci are known to be involved in dark pigmentation phenotype in the chicken breed Silkie (Bateson and Punnett, 1911; Dorshorst et al., 2010). The *Fm* locus is located on chromosome 20 and the causative mutation have been shown to be a complex rearrangement involving the *EDN3* gene (Dorshorst et al., 2011). The *id* locus is located at the Z chromosome. A region have been identified (Dorshorst et al., 2010; Siwek et al., 2013), but the gene have not yet been identified. A recent study on dermal shank pigmentation identified three significant SNPs in a 0.7 Mb region on (Li et al., 2014). Looking in detail at regions reported to contain these loci was therefore an obvious choice, although the Z chromosome was difficult to analyze correctly. A complicating factor is that both males and females of Hedemorahöna with light plumage color (such as wheaten, Columbian, salmon) rarely have very dark comb, i.e., even if they from the comb color in their offspring seem to have only the “dark allele” at the Z chromosome, their comb is not as dark as the birds with darker plumage colors but instead look more semi-dark. If they have completely white plumage color, they always have red comb, even if they have parents with dark comb (Thomas Englund, Pers. Commun.). This points to an interaction between the id locus and loci involved in plumage color.

The aim of this paper is to study genetic diversity in the two Swedish local chicken breeds Bohuslän-Dals svarthöna and Hedemorahöna. The now living birds in both of these breeds originate from small relicts of earlier larger populations. Here we map loci associated with the comb color trait that are segregating in within these breeds. To do this we genotyped 12 Bohuslän-Dals svarthöna and 22 Hedemorahöna with the 60k SNP chip produced by Illumina for the GWMAS Consortium (Groenen et al., 2011). An association study was performed on the genotypes given the recorded comb color phenotype.

## Material and methods

### Sampling and genotyping

The blood samples were collected by visits to the flocks by a veterinary student with special training for taking blood samples from chicken, or taken by a local veterinarian and sent to us. The blood sample was taken from the wing vein with a small needle and mixed with EDTA in an Eppendorf tube. Ethical permission (number C247/6) for the collection of blood samples was obtained from the Uppsala ethical board for animal research (name in Swedish: Uppsala djurförsöksetiska nämnd) prior to the collection of samples.

In this study 12 samples (three males and nine females) from Bohuslän-Dals svarthöna and 22 samples (six males and 16 females) from Hedemorahöna were included (Supplementary Table 1). The samples from Bohuslän-Dals svarthöna came from two different flocks and the samples from Hedemorahöna came from three different flocks.

For genotyping the 60k SNP chip, produced by Illumina for the GWMAS Consortium, was used (Groenen et al., 2011). The genotyping was done by the company DNA LandMarks in Canada. All positions given in this paper are from the SNP chip data. It is based on the genome assembly Gallus_gallus-2.1.

### Diversity analysis

The calculations of homozygosity of individuals and inbreeding were done with the function *het* in the program PLINK (Purcell et al., 2007, URL: http://pngu.mgh.harvard.edu/purcell/plink/) and were based on the autosomal markers (chromosomes 1–28). The analyses of fixation in the breeds were done by custom R scripts (available from the authors upon request).

### SNP quality control

Genome-wide analysis was done with GenAbel version 1.8-0 (Aulchenko et al., 2007). The data was filtered in GenAbel by iteratively excluding: markers with a call rate below 90%; markers with a minor allele frequency below 5%; markers that are not in Hardy-Weinberg equilibrium (*p* < 1 × 10^−8^) and individuals with a call rate below 85%. Additionally individuals with high autosomal heterozygosity (FDR <1%) and individuals with a pairwise Identity By State (IBS) value above 0.9 were removed. In total 34,955 markers (out of 53,312) and 31 individuals (out of 34) passed all the criteria.

### GWAS

A genome wide association was performed using the filtered markers and the comb color phenotype using the *qtscore* function in GenAbel. The comb color phenotype, measured for each individual, was encoded as 1, 2, and 3 (red, semi-dark, and dark, respectively). For each position on the genome an association analysis of the SNP genotype with the comb color phenotype was performed on all the individuals, as implemented in the *qtscore* function. Simultaneously population structure and relatedness in the data were corrected for. We calculated the genomic kinship matrix prior to the association analysis. The covariates, resulting from the relatedness, was subsequently calculated using the *polygenic* function and included by implementing the GRAMMAS method, in the *qtscore* function. The association of the comb color phenotype at each SNP included (and was thus corrected for) kinship and population effects. The analysis was permuted 10,000 times to obtain a FDR corrected, empirical genome wide significance.

To estimate the level of population stratification we performed a multidimensional scaling (MDS) analysis using the *cmdscale* function in R. This function was applied to the distances computed from the kinship matrix. We performed this analysis on the full dataset (i.e., both populations), as well as on the Hedemorahöna separately to check for substructure in this population.

## Results

### Diversity and inbreeding in the two breeds

The mean inbreeding coefficient (*F*) was considerably larger in the samples from Hedemorahöna than in the samples from Bohuslän-Dals svarthöna (Table 1). Most Bohuslän-Dals svarthöna individuals were not inbred, i.e., *F* were less than or close to 0 (Supplementary Table 1). The negative inbreeding coefficients show that these birds are more heterozygous than expected under Hardy–Weinberg equilibrium. However, half the Hedemorahöna individuals had *F* > 0.1 and most of these had *F* > 0.2 (Supplementary Table 1). The most inbred individual of Hedemorahöna had *F* = 0.45. The inbreeding varied between the three sampled flocks of Hedemorahöna with one flock having considerable higher inbreeding (mean *F* = 0.31) than the other two (mean *F* = 0.09 and 0.13, respectively). In the Bohuslän-Dals svarthöna, the difference between the flocks was smaller.

**Table 1:** **Description of the samples and result on inbreeding and homozygosity in the two breeds**.

	**Hedemorahöna**	**Bohuslän-Dals svarthöna**
Number of samples	22	12
Number of sampled flocks	3	2
Mean inbreeding coefficient (*F*)	0.17	−0.09
Mean number of homozygous SNPs in an individual	24,743	14,525
Comb color among samples	nine dark; two semi-dark; one red	two dark; 19 red; one unknown

There was a very large difference in homozygosity between the breeds. The number of homozygous SNPs in an individual was larger in Hedemorahöna (mean 24,743) than in Bohuslän-Dals svarthöna (mean 14,528). The most homozygous sample from Bohuslän-Dals svarthöna was much less homozygous than the least homozygous sample from Hedemorahöna.

In contrast to the large number of homozygous SNPs in each individual, our Hedemora samples have considerable more segregating SNPs within the breed than Bohuslän-Dals svarthöna (Table 2). In Hedemora about two thirds of the SNPs are segregating, whereas in Bohuslän-Dals svarthöna less than half of the SNPs are segregating. Twenty percent of the SNPs in the two breeds are fixed for the same allele.

**Table 2:** **Number of fixed and segregating SNPs on chromosomes 1–28 in the two breeds, both in the whole dataset and in 100 replicates of taking a subset of 12 Hedemorahöna at random to compare with the 12 Bohuslän-Dals svarthöna**.

	**Whole dataset (22 Hedemorahöna + 12 Bohuslän-Dals svarthöna)**	**Mean of 100 replicates of 12 random Hedemorahöna and all 12 Bohuslän-Dals svarthöna**
Genotypes present in both lines	50,617	50,615.27
Fixed for different alleles	767	970.14
Fixed for the same allele	10,326	10,965.43
Hedemorahöna fixed, Bohuslän-Dals svarthöna segregating	5583	6333.84
Bohuslän-Dals svarthöna fixed, Hedemorahöna segregating	16,166	15,322.71

**Figure 1:**
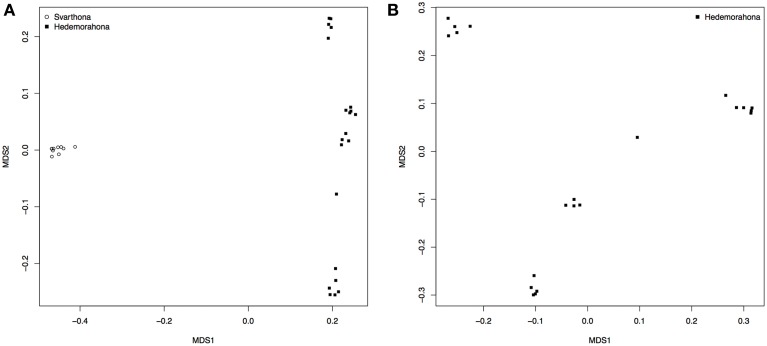
**Population stratification. (A)** MDS analysis on population indicating stratification by breed. **(B)** MDS on Hedemorahöna to estimate substructuring within this breed.

A multidimensional scaling plot shows that the two breeds form separate clusters well-separated from each other (Figure 1A). All the samples from Bohuslän-Dals svarthöna were close together, whereas the samples from Hedemorahöna was more spread out and showed some population structure (Figures 1A,B). Since the MDS-plots showed that there was clear differentiation between the two breeds (Figure 1A), we corrected for population structure in the association analyses.

### GWAS on autosomal markers in our data

**Figure 2:**
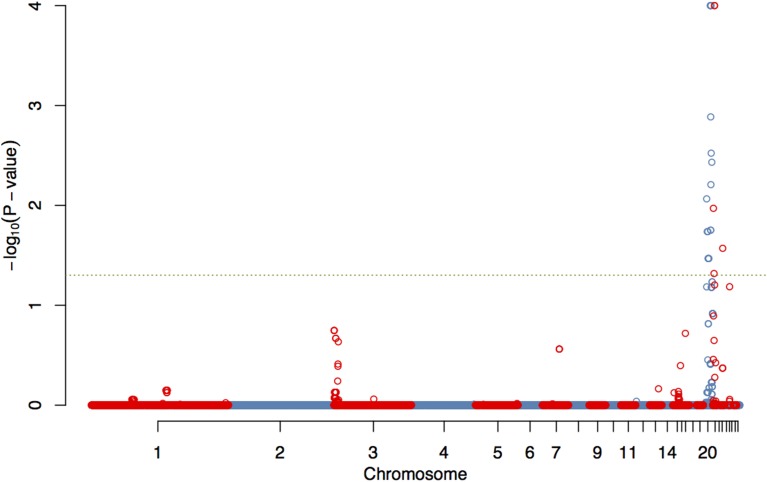
**Genome wide association of comb color phenotype**. Manhattan plot showing FDR-adjusted *p*-values for genome-wide associations of comb color, corrected for kinship.

**Figure 3:**
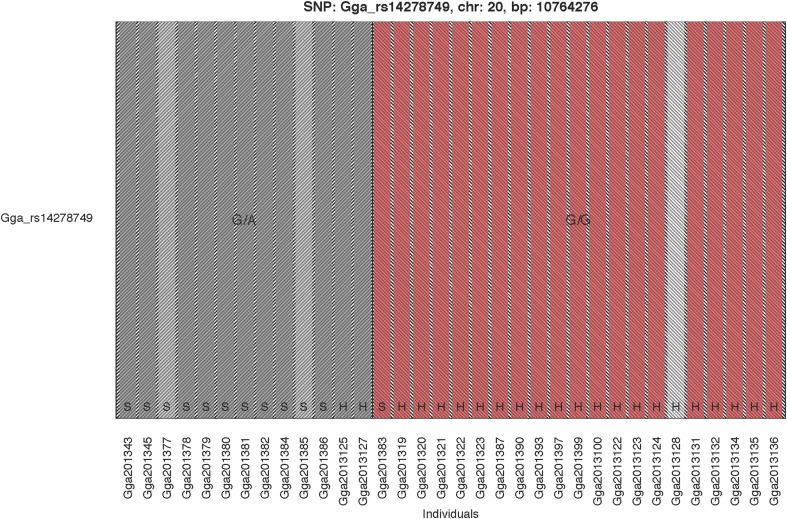
**Genotype Phenotype Map**. The genotype phenotype map for the SNP Gga_rs14278749 (position 10,764,276 on chromosome 20). Bar colors indicate comb color of individuals, with the white indicating the individual with unknown phenotype. The two shaded areas indicate the two observed genotypes, respectively. The breeds are indicated at the bottom of the bars with H and S for Hedemorahöna and Bohuslän-Dals Svarthöna, respectively.

**Figure 4:**
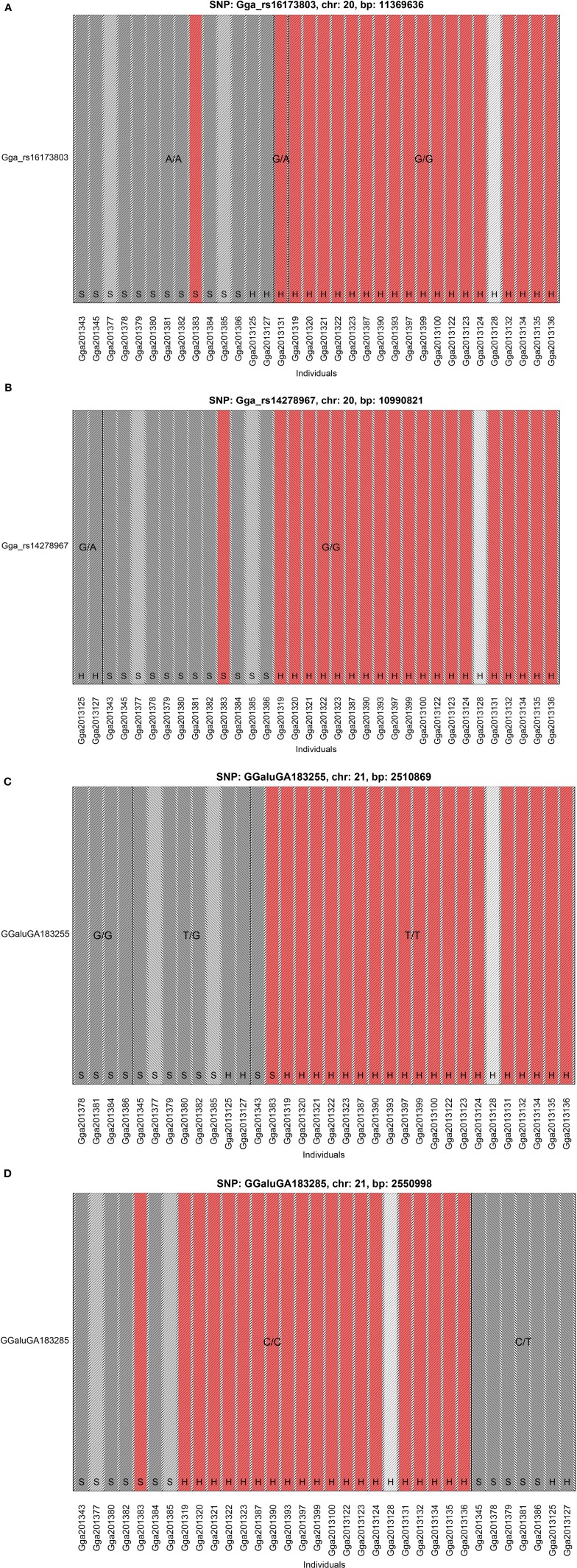
**Genotype Phenotype Maps**. The genotype phenotype maps for the SNPs **(A)** Gga_rs16173803 and, **(B)** Gga_rs14278967 on chromosome 20 as well as, **(C)** GGaluGA183255 and, **(D)** GGaluGA183285 on chromosome 21. Bar colors indicate comb color of individuals with, the white indicating the individual with unknown phenotype. Note that the two SNPs on chromosome 20 shown in this figure was not included in the GWAS, because they failed the quality control check. The reasons are that Gga_rs14278967 has a minor allele frequency of less than 5% while Gga_rs16173803 is not in Hardy-Weinberg-equilibrium (at *p*-level of 1e-08).

The GWAS on the two populations clearly identify regions on chromosomes 20 and 21 as associated with comb color (Figure 2, Supplementary Table 2). The associated markers on chromosome 20 are located between positions 10,194,286 and 12,375,897. This region encompasses the *EDN3* locus previously reported to be associated with dermal hyperpigmentation by Dorshorst et al. (2011). The SNP Gga_rs14278749 at position 10,764,276 on chromosome 20 was most significant (FDR *p* = 9.9 × 10^−5^). The genotype-phenotype map clearly shows that this SNP completely explain the red vs. dark comb color (Figure 3). Note that this SNP is located in the first duplicated region identified by Dorshorst et al. (2011). The interpretation of our genotype data should therefore be that the “heterozygotes” do have the duplication and that they have one allele in one copy of the segment and the other allele in the other copy of the segment. Since the duplication is segregating, the amount of heterozygotes was not high enough to make the SNP fail the quality control. On chromosome 21 the SNP GGaluGA183285 (FDR *p* = 9.9 × 10^−5^) and the SNP GGaluGA183255 (FDR *p* = 1.0 × 10^−04^) was most significant, these are located close to each other (position 2,550,998 and 2,510,869, respectively, Supplementary Table 2). Genotype–phenotype maps for these two SNPs on chromosome 21 can be seen in Figures 4C,D.

### Results on SNPs on chromosome 20 showing association with pigmentation in other studies

In the study by Dorshorst et al. (2011), the Silkie and Ayam Cemani samples were all heterozygous for five SNPs in the first duplicated region (positions 10,717,294–10,846,232) on chromosome 20. Among our 12 samples from Bohuslän-Dals svarthöna, all except one (which was the one with red comb) where heterozygous for two of these SNPs and two additional SNPs in this region. The two individuals from Hedemorahöna with dark comb were heterozygous for three of the five SNPs identified in Silkie and Ayam Cemani. The 11 samples from Bohuslän-Dals svarthöna with dark or semi-dark comb were also all heterozygous for three SNPs in the second duplicated region.

The SNP Gga_rs16173803 in the second duplicated region shows the same homozygous genotype in all the 12 samples from Bohuslän-Dals svarthöna and the two samples from Hedemorahöna with dark comb, one other Hedemora is heterozygous and all the rest are homozygous for the other allele (Figure 4A).

The SNP Gga_rs14278967 at position 10,990,821 at chromosome 20 is associated with Fm phenotype in Siwek et al. (2013). In our study, the two Hedemora with dark comb are heterozygous for this SNP, all other samples are homozygous for the same allele (Figure 4B). Note that also all genotyped individuals from Bohuslän-Dals svarthöna were homozygous for the major allele.

### Z chromosome

On chromosome Z, six markers in the region from position 107,457 to position 465,329 segregate perfectly together with the comb color phenotype in the breed Bohuslän-Dals svarthöna. The hen with the red comb have one allele while all the individuals with dark comb have the other allele. The two males with semi-dark comb are heterozygotes. This is not the same region on chromosome Z that has been shown to be associated with the id locus in other breeds previously (Dorshorst et al., 2010; Siwek et al., 2013). The SNP Gga_rs14685750 at position 72,985,598 at chromosome Z is associated with the id+/id+ genotype at Silkie and Green-legged Partridgelike fowl in Siwek et al. (2013). At this SNP, there is no variation in our samples from the Hedemorahöna and Bohuslän-Dals svarthöna. One adjacent SNP is fixed for one allele in Bohuslän-Dals svarthöna and another allele in Hedemorahöna, but many other such SNPs occur elsewhere on the Z chromosome. In Bohuslän-Dals svarthöna, several SNPs in this region have one allele in the red combed female and two dark combed females and the other allele in the other dark combed birds. The two semi dark males are heterozygous. In Hedemorahöna there is no variation in the SNPs closest to position 72,985,598. There are 21 SNPs showing a somewhat interesting pattern in this breed earlier on the chromosome (position 3,622,633–7,440,378). Here the two individuals with dark comb, the one with unknown comb color and one with red color have one allele and the rest of the individuals with red comb have the other allele. Interestingly, for these SNPs, the Bohuslän-Dals svarthöna is fixed for the dark comb allele.

## Discussion

Our results showed that the diversity, with respect to the breed, is larger in Hedemorahöna than in Bohuslän-Dals svarthöna. However, the individuals within Hedemorahöna have less diversity due to inbreeding.

A possibility is that the larger number of segregating SNPs in Hedemorahöna might explained by the fact that we had more samples from Hedemorahöna than from Bohuslän-Dals svarthöna. We therefore also analyzed the number of segregating SNPs in random subsets of 12 samples of Hedemorahöna. We replicated the random sampling 100 times and calculated the average number of fixed and segregating SNPs in the breeds over the replicates. The result was very similar to the results from the whole dataset (Table 2), strengthening the conclusion that there are much more segregating SNPs in Hedemorahöna.

As a comparison, Bohuslän-Dals svarthöna has about the same amount of segregating SNPs as the Low Line after 40 generations in the body weight selection experiment in Virginia (Johansson et al., 2010). The sample size of the Low line from the body weight selection experiment (20) is similar to our Hedemorahöna sample size (22).

Our GWAS and genotype–phenotype map confirmed that the region with the EDN3 gene (Fm) is associated with dark pigmentation in both breeds. The variation at a single SNP in this region completely explain if an individual has dark or red comb. However, the dark vs. semi-dark comb cannot be explained by this region. We also identified a region on chromosome 21 that is significantly associated with comb color. A possibility is that the region we identified on chromosome 21 together with id on the Z chromosome can explain if an individual have dark or semi-dark comb. However, our limited sample size with very few semi-dark individuals did not allow us to further explore this. With the model we used to correct for kinship in the GWAS it was not possible to analyze the Z chromosome. We also analyzed the data including the Z chromosome with a more simple model (data not shown), but did not find any significant results on the Z chromosome. We did find markers with interesting segregation patterns on the Z chromosome, although there were different markers in the two breeds. At the associated SNP in Siwek et al. (2013), there is no variation in any of our two breeds. The small sample size or lack of SNP variation might be the reason that we did not get any significant results on the Z chromosome, or our interesting patterns where some markers segregated with comb color in a breed might be just due to chance.

## Conclusion

In conclusion, we showed that the genetic diversity on the level of an individual is lower in our samples from Hedemorahöna than in Bohuslän-Dals svarthöna. In contrast, there is more diversity within the breed in Hedemorahöna than in Bohuslän-Dals svarthöna. The high inbreeding detected in many of our samples from Hedemorahöna is a cause for concern. When owners of this breed are getting a new breeding animal to their flock, they should aim to get an individual with low relatedness to the existing birds in the flock. We have shown here, using comb color as an example, that it is possible to map loci for traits segregating using a limited number of individuals, in these breeds. Regarding the comb color, we have shown that one locus on chromosome 20 (*EDN3*) determines if an individual will have red or dark comb, and that at least one other locus is probably involved in determining if a dark individual get a dark or semi dark comb.

### Conflict of interest statement

The authors declare that the research was conducted in the absence of any commercial or financial relationships that could be construed as a potential conflict of interest.
