# Supplementary material for: Characterization of genetic diversity and gene mapping in two Swedish local chicken breeds
Source: Front Genet. 2015 Feb 17;6:44. doi: 10.3389/fgene.2015.00044 (PMC4330917; doi:10.3389/fgene.2015.00044)
Supplement: Supplementary file 1 [file Table1.DOCX]

**Supplementary Table 1**. Information about the samples in this study

| **Sample** | **Breed** | **flock** | **Sex** | **Comb colour** | **F** |
| --- | --- | --- | --- | --- | --- |
| Gga201319 | Hedemorahöna | 1 | female | red | -0.04293 |
| Gga201320 | Hedemorahöna | 1 | female | red | 0.1843 |
| Gga201321 | Hedemorahöna | 1 | male | red | 0.1823 |
| Gga201322 | Hedemorahöna | 1 | female | red | 0.02625 |
| Gga201323 | Hedemorahöna | 1 | female | red | 0.09546 |
| Gga201387 | Hedemorahöna | 2 | female | red | 0.3584 |
| Gga201390 | Hedemorahöna | 2 | female | red | 0.05721 |
| Gga201393 | Hedemorahöna | 2 | male | red | 0.2989 |
| Gga201397 | Hedemorahöna | 2 | female | red | 0.4153 |
| Gga201399 | Hedemorahöna | 2 | male | red | 0.4488 |
| Gga2013100 | Hedemorahöna | 2 | male | red | 0.2884 |
| Gga2013122 | Hedemorahöna | 3 | female | red | 0.06108 |
| Gga2013123 | Hedemorahöna | 3 | female | red | -0.05684 |
| Gga2013124 | Hedemorahöna | 3 | female | red | 0.3685 |
| Gga2013125 | Hedemorahöna | 3 | female | dark | 0.08307 |
| Gga2013127 | Hedemorahöna | 3 | female | dark | 0.02583 |
| Gga2013128 | Hedemorahöna | 3 | female | unknown | 0.1998 |
| Gga2013131 | Hedemorahöna | 3 | female | red | 0.1265 |
| Gga2013132 | Hedemorahöna | 3 | female | red | 0.06129 |
| Gga2013134 | Hedemorahöna | 3 | female | red | 0.03606 |
| Gga2013135 | Hedemorahöna | 3 | male | red | 0.3094 |
| Gga2013136 | Hedemorahöna | 3 | male | red | 0.2166 |
| Gga201343 | Bohuslän-Dals svarthöna | 4 | female | dark | -0.1324 |
| Gga201345 | Bohuslän-Dals svarthöna | 4 | female | dark | 0.1115 |
| Gga201377 | Bohuslän-Dals svarthöna | 5 | male | semi dark | -0.1067 |
| Gga201378 | Bohuslän-Dals svarthöna | 5 | female | dark | -0.151 |
| Gga201379 | Bohuslän-Dals svarthöna | 5 | female | dark | -0.2729 |
| Gga201380 | Bohuslän-Dals svarthöna | 5 | female | dark | -0.2357 |
| Gga201381 | Bohuslän-Dals svarthöna | 5 | female | dark | 0.07062 |
| Gga201382 | Bohuslän-Dals svarthöna | 5 | female | dark | -0.4375 |
| Gga201383 | Bohuslän-Dals svarthöna | 5 | female | red | 0.009584 |
| Gga201384 | Bohuslän-Dals svarthöna | 5 | male | dark | 0.2027 |
| Gga201385 | Bohuslän-Dals svarthöna | 5 | male | semi dark | -0.1885 |
| Gga201386 | Bohuslän-Dals svarthöna | 5 | female | dark | 0.02574 |
