# Supplementary material for: Characterization of genetic diversity and gene mapping in two Swedish local chicken breeds
Source: Front Genet. 2015 Feb 17;6:44. doi: 10.3389/fgene.2015.00044 (PMC4330917; doi:10.3389/fgene.2015.00044)
Supplement: Supplementary file 2 [file Table2.DOCX]

**Supplementary Table 2.** SNPs significantly associated with comb colour correcting for population stratification and multiple testing (see also Figure 2).

| **SNP** | **Chromosome** | **Position** | **Allele 1** | **Allele 2** | **p-value** |
| --- | --- | --- | --- | --- | --- |
| Gga_rs14278749 | 20 | 11306226 | G | A | <0.0001 |
| Gga_rs15177670 | 20 | 10337508 | G | T | <0.0001 |
| Gga_rs16172014 | 20 | 10194286 | G | A | <0.0001 |
| Gga_rs14279248 | 20 | 11306226 | T | C | <0.0001 |
| GGaluGA183285 | 21 | 2550998 | C | T | 0.0001 |
| GGaluGA183255 | 21 | 2510869 | T | G | 0.0001 |
| Gga_rs15178088 | 20 | 10797631 | G | T | 0.0013 |
| Gga_rs16173979 | 20 | 11480890 | C | T | 0.0030 |
| Gga_rs16175291 | 20 | 12375897 | A | G | 0.0037 |
| GGaluGA180722 | 20 | 11061497 | G | A | 0.0062 |
| Gga_rs15172465 | 20 | 4853365 | C | A | 0.0086 |
| Gga_rs16177188 | 21 | 866101 | G | A | 0.0107 |
| GGaluGA180596 | 20 | 10717600 | C | T | 0.0177 |
| GGaluGA180605 | 20 | 10728799 | A | G | 0.0177 |
| Gga_rs16165971 | 20 | 5694375 | A | G | 0.0183 |
| Gga_rs14274441 | 20 | 6667990 | G | A | 0.0183 |
| GGaluGA188841 | 23 | 3692603 | G | T | 0.0269 |
| Gga_rs16167539 | 20 | 7035230 | C | T | 0.0340 |
| Gga_rs13633217 | 20 | 7071610 | G | A | 0.0340 |
| GGaluGA178691 | 20 | 7795012 | G | A | 0.0340 |
| Gga_rs16169020 | 20 | 8007281 | G | A | 0.0340 |
| Gga_rs15181258 | 21 | 1784095 | C | T | 0.0481 |
